# Late Pancreatic Metastasis From Papillary Thyroid Carcinoma Diagnosed by Endoscopic Ultrasound-Guided Tissue Acquisition

**DOI:** 10.31486/toj.24.0115

**Published:** 2025

**Authors:** César Vivian Lopes, Júlia Fernanda Semmelmann Pereira-Lima, Marianna Lins de Souza Salerno, Felipe Luzzatto

**Affiliations:** ^1^ Department of Gastroenterology and Digestive Endoscopy, Santa Casa Hospital, Porto Alegre, Brazil; ^2^ Department of Endocrinology, Santa Casa Hospital, Porto Alegre, Brazil; ^3^ Graduate Program in Pathology, Federal University of Health Sciences of Porto Alegre, Porto Alegre, Brazil; ^4^ Department of Pathology, Santa Casa Hospital, Porto Alegre, Brazil

**Keywords:** *Endoscopic ultrasound-guided fine needle aspiration*, *neoplasm metastasis*, *pancreatic neoplasms*, *thyroid cancer–papillary*

## Abstract

**Background:**

Papillary thyroid carcinoma, the most common differentiated thyroid cancer, has an indolent clinical course and a good prognosis. Metastases to the gastrointestinal tract account for <1% of all distant metastases, and the pancreas is an extremely rare site for metastasis from thyroid cancer.

**Case Report:**

We report the case of a patient who developed a pancreatic metastasis from a classic variant papillary thyroid carcinoma 11 years after total thyroidectomy, cervical lymphadenectomy, and radioactive iodine ablation. The patient experienced increased thyroglobulin levels, and abdominal computed tomography scan revealed a lesion in the uncinate process of the pancreas. Tissue samples obtained by endoscopic ultrasound-guided biopsy were positive for thyroglobulin and thyroid transcription factor 1. Because the patient was not a candidate for surgery, the metastatic lesion was not iodine-avid, and tyrosine kinase inhibitors could not be offered because of tumor-related symptoms, the patient was treated with stereotactic body radiotherapy only. The patient died almost 2 years after the diagnosis of metastatic papillary thyroid carcinoma to the pancreas (13 years after total thyroidectomy for the primary cancer).

**Conclusion:**

If pancreatic lesions are discovered during regular follow-up of patients who have previously been treated for papillary thyroid carcinoma, pancreatic metastasis must be considered, and imaging procedures other than whole-body iodine scintigraphy are required. Histopathology and iodine avidity will define the best therapeutic strategy. Radioactive iodine ablation should be considered for iodine-avid metastases, and surgery or tyrosine kinase inhibitors are promising options for non-iodine–avid lesions.

## INTRODUCTION

Differentiated thyroid cancers comprise the vast majority of thyroid malignancies, and the classic variant of papillary thyroid carcinoma is the most frequent type, with an indolent clinical course and good prognosis.^1^ Metastasis to distant organs occurs in <10% of patients, principally to lungs, bones, brain, and liver, but these metastases are a frequent cause of cancer-related mortality.^2^ Metastatic disease to the gastrointestinal tract is uncommon, accounting for <1% of all distant metastases.^3^ The pancreas is an extremely rare site for metastasis from thyroid cancer, with only 25 published case reports (involving 27 patients) in the English literature.^4-28^ Imaging alone cannot confirm the diagnosis; histopathologic analysis is critical for the definitive diagnosis of suspicious pancreatic lesions, especially in patients who have received previous treatment for other malignancies.^29^

We report the case of a patient who developed a pancreatic metastasis from a papillary thyroid carcinoma 11 years after undergoing total thyroidectomy and cervical lymphadenectomy for the primary thyroid cancer.

## CASE REPORT

A 68-year-old female with a 3.8-cm classic variant papillary thyroid carcinoma in the left lobe underwent total thyroidectomy with bilateral cervical lymphadenectomy in June 2011. The lesion invaded the parathyroid tissue, the resection margins were disease-free, and 2 of 5 regional lymph nodes were metastatic (pT2N1aM0) (American Joint Committee on Cancer tumor-node-metastasis [TNM] system),^30^ configuring the disease as stage II. Postoperatively, the patient received 150 mCi of iodine-131 radioactive iodine. Whole-body iodine scintigraphy demonstrated no uptake following radioactive iodine ablation, and stimulated thyroglobulin was 2.5 ng/mL (reference value, <2 ng/mL). The patient was monitored regularly, was asymptomatic, and had thyroglobulin levels <2 ng/mL for 9 years.

From October 2020 to March 2022, the patient's thyroglobulin levels progressively increased, with levels ranging from 2.5 to 51.7 ng/mL, but a metastasis screening with whole-body iodine scintigraphy showed no uptake in November 2020. In August 2021, cervical ultrasound showed no recurrence. In March 2022, bone scintigraphy showed abnormal uptake in the seventh thoracic vertebra, abdominal computed tomography (CT) scan revealed a 2.9 × 2.3-cm lesion in the uncinate process of the pancreas, and cervical ultrasound showed a 0.9 × 0.4-cm nodule close to the trachea. The patient's serum level of carbohydrate antigen 19-9 was normal (36.3 U/mL; reference range, 0-37 U/mL), but her thyroglobulin level was elevated at 35.7 ng/mL.

**Figure 1.**
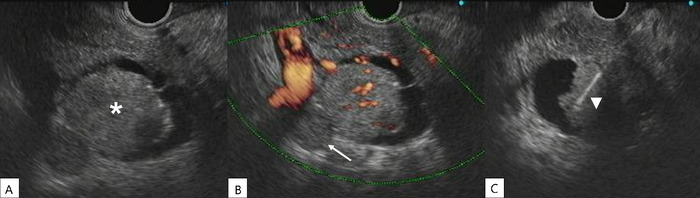
(A) Endoscopic ultrasound showed a well-circumscribed cystic lesion measuring 4.0 × 2.8 cm in the uncinate process and a mural nodule (asterisk) measuring 2.6 × 2.2 cm. (B) A neoplastic thrombus (arrow) was partially obstructing the superior mesenteric vein. (C) The mural nodule was punctured by endoscopic ultrasound-guided biopsy (arrowhead).

The patient was referred for endoscopic ultrasound evaluation and biopsy of the pancreatic lesion. A well-circumscribed cystic lesion (4.0 × 2.8 cm) with a mural nodule (2.6 × 2.2 cm) in the lumen was identified in the uncinate process (Figure 1). A neoplastic thrombus was next to the lesion, partially obstructing the superior mesenteric vein. Endoscopic ultrasound-guided biopsy was performed via the transduodenal approach. The tissue samples were processed as cell blocks for histologic and immunohistochemistry evaluation.

**Figure 2.**
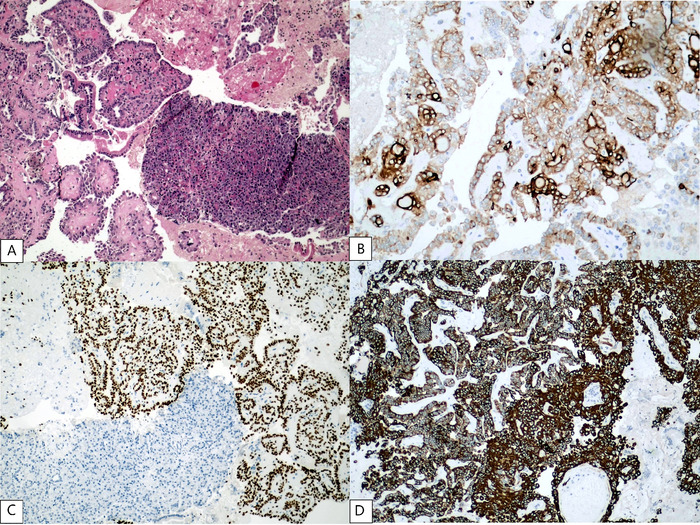
(A) Histologic findings of pancreatic metastasis from papillary thyroid carcinoma demonstrated a tumor consisting of cuboidal cells with irregular membrane, vacuolated cytoplasm, nuclear grooves, oval nuclei with granular chromatin, and no mitotic activity. A papillary arrangement and fibrovascular cores with pseudoinclusions were detected in the pancreatic tissue (hematoxylin and eosin stain, magnification ×400). Neoplastic cells showed intense immunoreactivity for (B) thyroglobulin (magnification ×200), (C) thyroid transcription factor 1 (magnification ×100), and (D) cytokeratin 7 (magnification ×100). Thyroglobulin and cytokeratin 7 were positive for membranous and cytoplasmic immunoreactivity. Thyroid transcription factor 1 indicates nuclear positivity.

Hematoxylin and eosin–stained sections demonstrated a hypercellular tumor consisting of cuboidal cells with irregular membrane, vacuolated cytoplasm, nuclear grooves, oval nuclei with granular chromatin, and no mitotic activity. The background was a papillary arrangement with complex randomly oriented papillae and fibrovascular cores, with pseudoinclusions in the pancreatic tissue. Immunohistochemical stains were positive for thyroglobulin, thyroid transcription factor 1, and cytokeratin 7. The histopathologic features and immunohistochemical profile confirmed a pancreatic metastasis from the classic variant papillary thyroid carcinoma (Figure 2). Our patient was not tested for the BRAF^V600E^ mutation.

**Figure 3.**
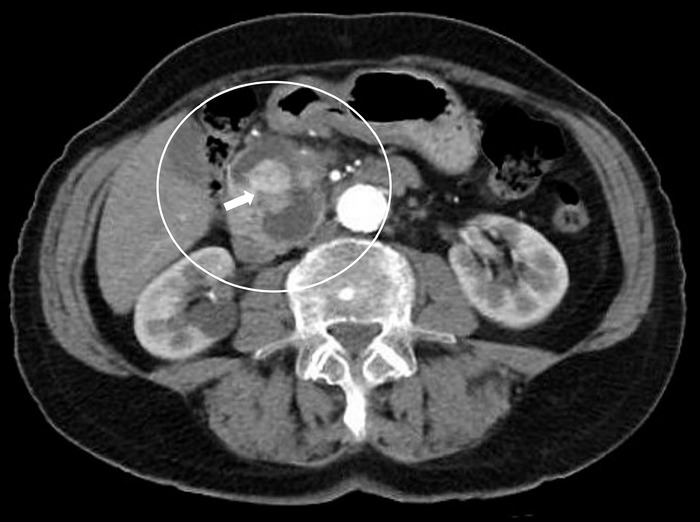
Contrast-enhanced axial abdominal computed tomography scan showed a 4.9 × 3.5 × 2.8-cm pancreatic cystic lesion in the uncinate process with a mural nodule in the cystic lumen (arrow).

In July 2022, whole-body 18-fluorodeoxyglucose positron emission tomography/computed tomography (^18^F-FDG-PET/CT) showed intense uptake in the pancreatic lesion, in 1 retroperitoneal lymph node, and in the seventh thoracic vertebra. Abdominal CT showed a retroperitoneal lymph node (1.1 × 1.1 cm) and an increase in the size of the pancreatic lesion (4.9 × 3.5 × 2.8 cm) (Figure 3). The patient's thyroglobulin level was 35.7 ng/mL.

Pancreatic resection was not considered because of multiorgan metastases. The patient was treated with 20 Gy of stereotactic body radiotherapy. Specifically for bone metastasis, the patient received a single dose of 18 Gy of external beam radiotherapy and denosumab 120 mg, 1 ampoule subcutaneously per month from September 2022 to September 2023. In September 2022, the patient received a second round of radioactive iodine ablation with 150 mCi of iodine-131. Whole-body iodine scintigraphy following radioactive iodine ablation showed no iodine-avid metastases. The patient's serum thyroglobulin level decreased to 23.2 ng/mL.

In March 2023, a repeat whole-body ^18^F-FDG-PET/CT revealed stability of the intense uptake in the pancreatic lesion, progression of the size and uptake in the retroperitoneal lymph node, and partial decrease of the uptake in the seventh thoracic vertebra. Abdominal CT showed the pancreatic lesion was stable in size and confirmed the enlargement of the retroperitoneal lymph node (3.0 × 2.0 cm). For the lymph node, the patient received a total of 35 Gy of stereotactic body radiotherapy.

In September 2023, abdominal CT showed several liver metastases, but the sizes of the pancreatic lesion (4.6 × 4.2 cm) and the retroperitoneal lymph node (3.1 × 3.0 cm) were stable. Until December 2023, despite the radiologic findings, the patient was asymptomatic and clinically doing very well.

However, in January 2024, the patient was hospitalized with abdominal pain, high fever, jaundice, and cholangitis as a result of progression of liver metastases and bile duct obstruction. Endoscopic retrograde cholangiopancreatography was performed for prosthesis placement and relief of the obstruction. Abdominal CT showed an increased number of liver lesions (the largest measured 3.0 cm), the pancreatic lesion had enlarged (5.3 × 4.5 cm), and the retroperitoneal lymph node was stable.

In February 2024, the patient's clinical condition notably worsened. Tyrosine kinase inhibitors could not be administered because of the patient's multiple tumor-related symptoms. Only supportive care could be offered. The patient's last thyroglobulin level was 139 ng/mL, and she died 23 months after the diagnosis of metastatic papillary thyroid carcinoma to the pancreas, which was 13 years after total thyroidectomy for the primary cancer.

## DISCUSSION

Including our case, only 26 reports (involving 28 patients with a total of 29 lesions) of primary papillary thyroid carcinomas with pancreatic metastasis have been reported to date (Tables 1 and 2).^4-28^

**Table 1.** Patient and Disease Details From Reports of Primary Papillary Thyroid Carcinomas With Pancreatic Metastasis

Study	Age, years	Sex	Histology	TNM Classification	Stage	Thyroidectomy	RAI	Locoregional Recurrence	Metastases Before Pancreatic Metastasis
Sugimura et al, 1991^4^	32	F	Classic	NR	NR	Total	NR	NR	NR
Jobran et al, 2000^5^	52	M	Tall cell	T4aN1M0	I	Total	Yes	Yes	No
Meyer and Behrend, 2006^6^	62	M	Classic	T4aN0M0	III	Total	Yes	Yes	Lung, adrenal
Siddiqui et al, 2006^7^	62	M	Tall cell	T4aN1bM0	III	Total	Yes	No	Lung
Angeles-Angeles et al, 2009^8^	72	M	Classic	T4bNxM1	IVB	None	Yes	No	No
Chen and Brainard, 2010^9^	77	M	Classic	T3N1M0	II	Total	Yes	Yes	No
Borschitz et al, 2010^10^	34	F	Follicular	T3N1aM0	I	Total	NR	Yes	No
	46	M	Classic	T3N1M0	I	Total	Yes	Yes	No
Alzahrani et al, 2012^11^	47	M	Classic	T4aN1bM0	I	Total	Yes	Yes	Lung
Olson et al, 2013^12^	59	F	Classic	NR	NR	NR	NR	NR	No
Tunio et al, 2013^13^	60	M	Follicular	T2N1M0	II	Total	Yes	No	No
Li et al, 2014^14^	55	M	Classic	NR	NR	Partial	NR	Yes	No
Davidson et al, 2017^16^	82	F	Tall cell	T3N1bM0	II	Total	Yes	Yes	No
Murakami et al, 2018^17^	66	F	Classic	T4aN1bM0	III	Total	NR	Yes	No
Cho et al, 2019^15^	71	M	Classic	NR	NR	Total	Yes	Yes	Lung
Yang et al, 2019^19^	48	M	Classic	T4aN1M1	II	Total	Yes	No	No
Tramontin et al, 2020^18^	67	M	Classic	T4aN1bM0	III	Total	Yes	Yes	No
Ren et al, 2020^20^	47	M	Classic	T4aN0M1	II	Total	Yes	No	No
Rossi et al, 2020^21^	45	M	Classic	NR	NR	NR	NR	Yes	Lung, brain
Uslar et al, 2020^22^	80	M	Tall cell	T4aN1M1	IVB	Total	Yes	No	No
Yoon et al, 2020^23^	71	M	Classic	T3NxM0	II	NR	NR	NR	NR
	61	M	Classic	T3N1bM0	II	NR	NR	NR	NR
Stein et al, 2020^24^	46	M	Tall cell	T4aN1M0	I	Total	Yes	Yes	No
Wong et al, 2021^25^	68	M	Classic	NR	NR	NR	Yes	NR	NR
Warda et al, 2022^26^	46	M	Tall cell	T4aN1M0	I	Total	Yes	Yes	No
Song et al, 2023^27^	51	M	Classic	T3N1bM0	I	Total	Yes	Yes	No
Ahmadian et al, 2024^28^	66	M	Classic	NR	NR	Total	Yes	NR	NR
Present case	79	F	Classic	T2N1M0	II	Total	Yes	No	No

F, female; M, male; NR, not reported; RAI: radioactive iodine ablation; TNM, tumor-node-metastasis.

**Table 2.** Diagnostic, Treatment, and Outcome Details of Pancreatic Metastases From Reports of Patients With Papillary Thyroid Carcinomas

Study	Time After PTC Diagnosis, years	Clinical Features	Imaging	Iodine- Avid	Local	Size, cm	Solid/Cyst	Sampling	Other Metastases at Diagnosis of PM	Treatment	Follow-up, months	Outcome
Sugimura et al, 1991^4^	7	Jaundice, itching	CT, US	NR	Head	3	NR	NR	No	Surgery (PD)	NR	NR
Jobran et al, 2000^5^	1	Abdominal pain	MRI	No	Head	3.5/2.5	Cyst	CT-FNA	Lung, vertebra	Surgery (PD), carboplatin and doxorubicin	0.5	Died
Meyer and Behrend, 2006^6^	5	Anemia, duodenal bleeding ulcer	CT	No	Head	NR	Cyst	Endoscopic biopsy of duodenal ulcer	Lung, liver, kidney	Surgery (DP)	54	Died
Siddiqui et al, 2006^7^	7	Abdominal pain	CT, PET	No	Head	1.5	Solid	EUS-TA	Lung	Surgery (PD)	24	Alive
Angeles-Angeles et al, 2009^8^	Synchronous	Abdominal pain	CT	NR	Tail	8.5	Solid	Upfront surgery	Brain, vertebra	Surgery (DP)	8	Alive
Chen and Brainard, 2010^9^	5	Pancreatitis	EUS	NR	Neck	3	Solid	EUS-TA	NR	NR	NR	NR
Borschitz et al, 2010^10^	10	NR	CT, PET, MRI	No	Head	4	Solid	Upfront surgery	No	Surgery (DP)	7	Alive
	15	NR	CT, PET	No	Body	NR	Solid	Upfront surgery	Lung, bone, skin	Surgery (enucleation), RAI	36	Died
Alzahrani et al, 2012^11^	8	Asymptomatic	PET, MRI	No	UP	1.7	Solid	EUS-TA	Lung, vertebra, liver, peritoneum	Sorafenib	20	Died
Olson et al, 2013^12^	Synchronous	NR	NR	NR	NR	NR	NR	EUS-TA	NR	NR	NR	NR
Tunio et al, 2013^13^	7	Abdominal pain	CT, MRI	No	Neck	1.8	Solid	CT-FNA	Lung	Surgery (PD), sorafenib	36	Alive
Li et al, 2014^14^	11	Asymptomatic	CT, PET	NR	Body/tail	6.2	Solid	Upfront surgery	No	Surgery (DP)	5	Died
Davidson et al, 2017^16^	2	Asymptomatic	CT, PET	NR	Body	1.1	Solid	EUS-TA	No	Surveillance	6	Alive
Murakami et al, 2018^17^	14	Asymptomatic	CT, PET, MRI	NR	Body	3	Solid	EUS-TA	Lung	Surgery (DP), paclitaxel	11	Died
Cho et al, 2019^15^	10	Asymptomatic	PET, MRI	NR	Body	1.4	Solid	EUS-TA	Lung	Surveillance	1	Alive
Yang et al, 2019^19^	Synchronous	Abdominal pain	US	NR	Body	2.5	Solid	Upfront surgery	Lung, liver, kidney, parotid, skin, skeletal muscle, bone	Surgery (DP), lenvatinib	18	Alive
Tramontin et al, 2020^18^	6	Jaundice, nausea, weight loss	PET	NR	Head	3.5	Solid	EUS-TA	No	Surgery (PD), sorafenib	7	Alive
Ren et al, 2020^20^	Synchronous	Abdominal pain	CT, US	NR	Body	4	Solid	Upfront surgery	Liver, diaphragm, parotid	Surgery (DP), RAI	5	Alive
Rossi et al, 2020^21^	15	Pancreatitis	CT, MRI	NR	Head	2	Solid	EUS-TA	No	Surgery (PD)	NR	NR
Uslar et al, 2020^22^	Synchronous	Jaundice	CT, PET, MRI,	NR	Head	3.6	Solid	EUS-TA	Lung, brain, skeletal muscle	RAI and RT	12	Alive
Yoon et al, 2020^23^	NR	NR	CT, PET	NR	Head	6	Solid	EUS-TA	Lung	Lenvatinib	36	Alive
	NR	Asymptomatic	NR	NR	Body/tail	7	NR	CT-FNA	Lung, bone	Lenvatinib	23	Alive
Stein et al, 2020^24^	3	NR	PET	No	Head	3	Solid	Upfront surgery	Vertebra	Surgery (PD), lenvatinib	6	Alive
Wong et al, 2021^25^	7	NR	PET	Yes	Body/tail	NR	Solid	NR	NR	RAI	NR	NR
Warda et al, 2022^26^	3	NR	PET	NR	Head	3	NR	Upfront surgery	No	Surgery (PD), lenvatinib	NR	Alive
Song et al, 2023^27^	6	Diabetes	CT, PET, MRI	NR	Tail	5.6	Cyst	Upfront surgery	No	Surgery (DP)	36	Alive
Ahmadian et al, 2024^28^	5	Asymptomatic^a^	CT, PET	NR	Tail	NR	Solid	EUS-TA	Liver, adrenal, peritoneum	NR	NR	NR
Present case	11	Asymptomatic	CT, PET	No	UP	4	Cyst	EUS-TA	Liver, vertebra, retroperitoneal lymph node	RAI, RT	23	Died

^a^The patient had pneumonia but no tumor-related symptoms.

Note: All pancreatic metastases were solitary lesions, except for a single patient with 2 lesions in the pancreatic head.^5^

CT, computed tomography; CT-FNA, computed tomography-guided fine needle aspiration; DP, distal pancreatectomy; EUS, endoscopic ultrasound; EUS-TA, endoscopic ultrasound-guided tissue acquisition; MRI, magnetic resonance imaging; NR, not reported; PD, pancreaticoduodenectomy; PET, positron emission tomography; PM, pancreatic metastasis; PTC, papillary thyroid carcinoma; RAI, radioactive iodine ablation; RT, radiotherapy; UP, uncinate process; US, ultrasound.

Among the 28 patients, there was a preponderance of males (22 cases), and the median age of patients at diagnosis of pancreatic metastasis was 66 years (range, 39-84 years). Papillary thyroid carcinoma staging was available for 21 cases, 15 of which were stages I (n=7) and II (n=8) and 6 of which were stages III (n=4) and IVB (n=2). In most of the cases, papillary thyroid carcinoma was treated with thyroidectomy and radioactive iodine ablation. After surgery, locoregional recurrence occurred in 15 of 22 (68.2%) patients. Only 4 recurrent cases presented distant metastases prior to the pancreatic metastases, with the lungs affected in all 4 cases. Another case reported by Siddiqui et al involved a patient who presented with lung metastasis before the occurrence of pancreatic metastasis, but this case did not represent locoregional recurrence of thyroid cancer.^7^ With the exclusion of 5 synchronous lesions (ie, pancreatic metastasis detected at papillary thyroid carcinoma presentation), the median latency for pancreatic metastasis was 7 years, ranging from 1 to 15 years after papillary thyroid carcinoma treatment. The latency period was >5 years for 66.7% (14/21) of patients, with a median of 9 years.

Pancreatic metastases were detected by CT and/or PET scan in 17 cases. Both imaging modalities were used in 11 patients, with 4 patients also receiving magnetic resonance imaging (MRI). PET scan and CT scan were the single imaging procedure for 4 and 2 patients, respectively. Eight patients received CT and/or PET with MRI. Two patients received CT and ultrasound. The median size of the pancreatic metastases was 3 cm (range, 1.1-8.5 cm), and the median size of the pancreatic metastases for symptomatic and asymptomatic patients was the same (3 cm). The lesions of symptomatic patients were principally in the pancreatic head (7/13, 53.8%), while the lesions of asymptomatic patients were primarily in the body and tail (6/8, 75%). All pancreatic metastases were solitary lesions, except for a single patient with 2 lesions in the pancreatic head.^5^

Twenty metastases were solid with well-defined borders, and only 3 previously reported lesions presented a cystic area,^5,6,27^ as in our case. The uncinate process, head, and neck were involved in 15 cases, the body and/or tail in 12 cases, and the location was not reported for 1 case. Clinical features were reported for 21 patients. Of these patients, 13 were symptomatic: 8 had abdominal pain, and 2 of these patients had pancreatitis. Seventeen patients had other distant metastases at the diagnosis of pancreatic metastasis. Pulmonary metastases were the most common distant metastases (occurring in 12 cases), and bone metastases affected 8 patients, with 5 cases compromising vertebrae. Both lung and bone metastases were concurrent in 5 cases.

In 3 of 5 synchronous lesions, pancreatic metastases were detected before the primary papillary thyroid carcinoma.^8,19,20^ These lesions were located in the body or tail, and the leading complaint was abdominal pain. These 3 patients underwent surgery, and other distant metastases were found shortly before or after resection. After surgery, these patients were alive after a median follow-up of 8 months (range, 5-18 months).

After total thyroidectomy with or without radioactive iodine ablation, patients with papillary thyroid carcinoma may present with elevated thyroglobulin levels, revealing the presence of thyroid tissue in the body that indicates either residual tissue or locoregional/distant metastases.^31^ Metastatic lesions can be iodine-avid or not. A patient may have elevated thyroglobulin levels even in the presence of non-iodine–avid metastases. These lesions will not be identified by whole-body iodine scintigraphy because they do not demonstrate any iodine uptake.^32^

Because pancreatic imaging alone cannot ensure the detection of pancreatic metastasis, histopathologic evaluation of any pancreatic lesion in patients with previous malignancy is mandatory for definitive diagnosis.^29^ Samples for histopathology were obtained before surgery in 17 of the reported cases, 13 of them, including our case, by endoscopic ultrasound-guided tissue acquisition.^7,9,11,12,15-18,21-23,28^ Nine other patients underwent surgery with no previous biopsy.^8,10,14,19,20,24,26,27^

Metastatic thyroid cancer is immunohistochemically defined by a positive finding for thyroglobulin and thyroid transcription factor 1.^33^ Including our case, immunohistochemical studies were positive for thyroglobulin in 18 cases^5-11,14,16,17,19,20,22,24,26,27^ and for thyroid transcription factor 1 in 17 cases.^7,8,11,14-22,24,26-28^ Thirteen cases were positive for both markers.^7,8,11,14,16,17,19,20,22,24,26,27^ Information on both markers was not reported in 6 cases (Table 3).

**Table 3.** Immunohistochemistry and Genetic Findings of Pancreatic Metastases From Reports of Patients With Papillary Thyroid Carcinomas

Study	Thyroglobulin	Thyroid Transcription Factor 1	BRAF^V600E^ Mutation
Sugimura et al, 1991^4^	NR	NR	NR
Jobran et al, 2000^5^	Positive	NR	NR
Meyer and Behrend, 2006^6^	Positive	NR	NR
Siddiqui et al, 2006^7^	Positive	Positive	NR
Angeles-Angeles et al, 2009^8^	Positive	Positive	NR
Chen and Brainard, 2010^9^	Positive	NR	NR
Borschitz et al, 2010^10^	Positive	NR	Negative
	Positive	NR	Positive
Alzahrani et al, 2012^11^	Positive	Positive	Positive
Olson et al, 2013^12^	NR	NR	NR
Tunio et al, 2013^13^	NR	NR	Positive
Li et al, 2014^14^	Positive	Positive	NR
Davidson et al, 2017^16^	Positive	Positive	Positive
Murakami et al, 2018^17^	Positive	Positive	Positive
Cho et al, 2019^15^	NR	Positive	NR
Yang et al, 2019^19^	Positive	Positive	Positive
Tramontin et al, 2020^18^	NR	Positive	NR
Ren et al, 2020^20^	Positive	Positive	Positive
Rossi et al, 2020^21^	NR	Positive	NR
Uslar et al, 2020^22^	Positive	Positive	Positive
Yoon et al, 2020^23^	NR	NR	Negative
	NR	NR	NR
Stein et al, 2020^24^	Positive	Positive	NR
Wong et al, 2021^25^	NR	NR	NR
Warda et al, 2022^26^	Positive	Positive	Positive
Song et al, 2023^27^	Positive	Positive	Positive
Ahmadian et al, 2024^28^	NR	Positive	Negative
Present case	Positive	Positive	NR

NR, not reported.

The ideal therapeutic approach for treating pancreatic metastasis from papillary thyroid carcinoma is unknown. Surgical resection was the most frequent treatment in the reported cases (17 patients), including 8 pancreati-coduodenectomies,^4,5,7,13,18,21,24,26^ 8 distal pancreatecto-mies,^6,8,10,14,17,19,20,27^ and 1 enucleation.^10^ Ten patients with other concurrent metastases underwent surgery.^5-8,10,13,17,19,20,24^ Among these patients, 7 had pulmonary metastases, 5 patients had bone metastases, and 3 patients had concomitant lung and bone metastases.

In the presence of iodine-avid metastases, radioactive iodine ablation followed by thyroid-stimulating hormone suppression with thyroxine is the mainstay of metastatic disease treatment.^31^ Simões-Pereira et al investigated the differences in radioactive iodine avidity and outcome among specific histotypes of metastatic differentiated thyroid cancer and reported that iodine avidity in metastases occurred in 21.4% of patients with classic variant papillary thyroid carcinoma.^32^ The outcome of iodine-avid metastases from classic variant papillary thyroid carcinoma after radioactive iodine ablation was stable disease or partial/complete response in 55.6% of patients, with a progression-free survival of 32 months. For patients with classic variant papillary thyroid carcinoma, 5- and 10-year disease-specific survival was 69.2% and 53.3%, respectively. In their analysis of the outcomes of non-iodine–avid patients, Simões-Pereira et al found that 72.7% of patients with classic variant papillary thyroid carcinoma had evidence of progressive metastatic disease after radioactive iodine treatment.^32^ Our patient had a non-iodine–avid pancreatic metastasis, and whole-body iodine scintigraphy demonstrated no pancreatic uptake following a second round of radioactive iodine ablation with 150 mCi of iodine-131. Including our case, 5 patients in the reported cases were treated with radioactive iodine ablation,^10,20,22,25^ with a single patient receiving radioactive iodine ablation as the only treatment.^25^

When whole-body iodine scintigraphy shows no uptake in metastatic sites, tyrosine kinase inhibitors are an option for treatment of radioiodine-refractory metastases. In their systematic review and meta-analysis, Yu et al showed that the use of tyrosine kinase inhibitors significantly improved progression-free survival, and some tyrosine kinase inhibitors increased overall survival, although with a high incidence of side effects.^34^ A meta-analysis by Thomas et al that included 219 patients treated with sorafenib for metastatic thyroid cancer reported a partial response in 20.9%, stable disease in 58.3%, and progressive disease in 20.9% of patients with differentiated thyroid cancer.^35^ No study reported complete responses. In our review, 8 patients received tyrosine kinase inhibitors, either sorafenib or lenvatinib. Among the patients treated with sorafenib,^11,13,18^ 2 of 3 patients^13,18^ received the drug after surgery and had stable disease. The patient who received sorafenib as the sole treatment had progressive disease.^11^

Lenvatinib results have been more favorable. In the experience reported by Masaki et al with 42 patients, partial response was observed in 26 (62%) patients, stable disease in 10 (24%) patients, and progressive disease in 6 (14%) patients treated with lenvatinib.^36^ The overall survival of all treated patients was 51% at 3 years.^36^ In 4 cases reporting 5 pancreatic metastases from papillary thyroid carcinoma that were treated with lenvatinib,^19,23,24,26^ 2 patients who did not undergo resection had a partial response to treatment,^23^ and 3 patients who underwent resection did not develop recurrence or new pancreatic metastases.^19,24,26^ The presence of tumor-related symptoms prior to tyrosine kinase inhibitor use or symptom occurrence during treatment is an independent predictor of poor clinical benefit and poor survival.^37^ For this reason, our patient could not receive treatment with tyrosine kinase inhibitors.

In our review, the overall median follow-up for patients with pancreatic metastases from papillary thyroid carcinoma was 12 months. Patients who did not undergo surgery had a longer median follow-up period compared to patients who underwent surgery (20 months vs 9.5 months, respectively). Overall mortality was 31.8%, and the mortality rate was similar for patients who underwent surgery vs patients who did not undergo surgery (33.3% vs 28.6%, respectively). These findings must be interpreted cautiously because we only evaluated case reports, most of which involved a single patient, and several case reports were missing data.

Some mutations occur frequently in papillary thyroid carcinoma. BRAF^V600E^ is the most common oncogene in papillary thyroid carcinoma, with an average prevalence between 25% to 89.2%.^38,39^ Compared with wild-type BRAF, the BRAF^V600E^ mutation is associated with more aggressive features, including extrathyroidal invasion, lymph node metastasis, advanced TNM stage, recurrence, and decreased survival.^39^ Although the association between the BRAF^V600E^ mutation and distant metastases is still controversial,^38,39^ positive BRAF^V600E^ metastases are frequently non-iodine–avid lesions with higher ^18^F-FDG avidity.^40,41^ In our review, the status of the BRAF^V600E^ mutation was evaluated in 13 cases, and the mutation was present in 10 (76.9%) patients (Table 3). In the patients presenting with the mutation, papillary thyroid carcinoma stage was I (4) and II (4) for 8 cases, and locoregional recurrence occurred in 6/10 (60%) cases. The median size of pancreatic metastases was 3 cm, 5 of 8 (62.5%) cases were symptomatic, and 7/10 (70%) cases had concurrent metastases. Mortality was 30% (3/10). Because the BRAF^V600E^ mutation was analyzed in fewer than half the cases in our review, we cannot draw definitive conclusions about the importance of the mutation on the prognosis of these patients.

## CONCLUSION

Our patient developed a pancreatic metastasis from a classic variant papillary thyroid carcinoma more than a decade after thyroidectomy. Although rare, pancreatic metastasis must be considered and requires imaging procedures other than whole-body iodine scintigraphy during regular follow-up of patients with prior malignancies. If a lesion is detected, histopathologic evaluation is mandatory. Because best management for these cases has not yet been defined, radioactive iodine ablation should be considered for iodine-avid lesions, and surgery or tyrosine kinase inhibitor therapy are promising for non-iodine–avid metastases.

## Notes

*This article meets the Accreditation Council for Graduate Medical Education and the American Board of Medical Specialties Maintenance of Certification competencies for Patient Care and Medical Knowledge.*
